# Ceruloplasmin and hephaestin jointly protect the exocrine pancreas against oxidative damage by facilitating iron efflux

**DOI:** 10.1016/j.redox.2018.05.013

**Published:** 2018-05-31

**Authors:** Min Chen, Jiashuo Zheng, Guohao Liu, En Xu, Junzhuo Wang, Brie K. Fuqua, Chris D. Vulpe, Gregory J. Anderson, Huijun Chen

**Affiliations:** a Jiangsu Key Laboratory of Molecular Medicine, Medical School of Nanjing University, China; b Center for Environmental and Human Toxicology, Department of Physiological Sciences, University of Florida, Gainesville, FL, USA; c QIMR Berghofer Medical Research Institute, Brisbane, Queensland, Australia

**Keywords:** MCF, multicopper ferroxidase, HEPH, hephaestin, CP, ceruloplasmin, ROS, reactive oxygen species, FPN1, ferroportin1, GPI, glycosylphosphatidylinositol, KO, knockout, WT, wild-type, PBS, phosphate buffered saline, IRE, iron responsive element, SOD, superoxide dismutase, GPx, glutathione peroxidase, MDA, malondialdehyde, Multicopper ferroxidase, Hephaestin, Ceruloplasmin, Pancreas, Oxidative damage, Iron efflux

## Abstract

Little is known about the iron efflux from the pancreas, but it is likely that multicopper ferroxidases (MCFs) are involved in this process. We thus used hephaestin (*Heph*) and ceruloplasmin (*Cp*) single-knockout mice and *Heph/Cp* double-knockout mice to investigate the roles of MCFs in pancreatic iron homeostasis. We found that both HEPH and CP were expressed in the mouse pancreas, and that ablation of either MCF had limited effect on the pancreatic iron levels. However, ablation of both MCFs together led to extensive pancreatic iron deposition and severe oxidative damage. Perls’ Prussian blue staining revealed that this iron deposition was predominantly in the exocrine pancreas, while the islets were spared. Consistent with these results, plasma lipase and trypsin were elevated in *Heph/Cp* knockout mice, indicating damage to the exocrine pancreas, while insulin secretion was not affected. These data indicate that HEPH and CP play mutually compensatory roles in facilitating iron efflux from the exocrine pancreas, and show that MCFs are able to protect the pancreas against iron-induced oxidative damage.

## Graphical abstract

**Figure:**
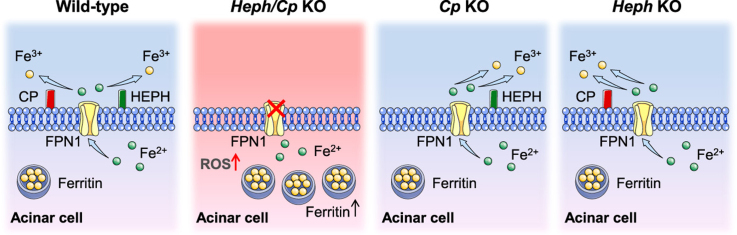

## 1 Introduction

Iron is a redox active metal that can exist in the ferrous or ferric state, and it is essential for many basic physiological processes [1]. However, excess ferrous iron can generate toxic reactive oxygen species (ROS) that can damage proteins, lipids, and DNA [2]. The safe handling of iron in the body is maintained by a complex interaction among multiple iron-binding proteins, transporters, receptors, ferroxidases, and ferrireductases [3], [4]. Disruption of this finely-tuned system can lead to iron overload or iron deficiency and associated adverse effects [2], [4].

Multicopper ferroxidases (MCFs) are known to facilitate cellular iron efflux in conjunction with the membrane ferrous iron exporter ferroportin1 (FPN1), by oxidizing ferrous iron to the ferric state [1]. Three MCFs, namely ceruloplasmin (CP), hephaestin (HEPH), and zyklopen, have been identified in vertebrates [1], [5]. Despite their similar function, these MCFs vary widely in tissue distribution. HEPH is expressed most strongly in the small intestine and, accordingly, mice with global *Heph* knockout display severe iron accumulation in duodenal enterocytes [6]. However, HEPH is also expressed in human pancreatic *β*-cells [7] and a range of other tissues. CP is largely recognized as a soluble serum protein which is secreted by the liver, but it has also been found as a glycosylphosphatidylinositol (GPI)-linked protein in astrocytes and multiple organs [8], [9]. Individuals with mutations in the *CP* gene (aceruloplasminemia) display significant iron deposition in the liver, glial cells, and, interestingly, in the pancreas [10].

The pancreas can easily become iron-overloaded. Patients with hereditary hemochromatosis, a common inherited disorder characterized by excessive dietary iron absorption and iron accumulation in many tissues, show most extensive iron deposition in the liver, heart, and pancreas [11]. Furthermore, iron accumulation in the exocrine pancreas has been found in a series genetically engineered mouse models that are characterized by systemic iron loading, including hypotransferrinaemic mice, and bone morphogenetic protein 6-, hemojuvelin-, and hepcidin-deficient strains [12], [13], [14], [15]. These studies suggest that the pancreas is very active in taking up iron from the plasma. They also imply that the pancreas needs an equally efficient iron efflux mechanism to balance iron levels and protect itself against oxidative damage. Although FPN1 is expressed in human pancreatic islets and ductal epithelial cells [7] and in mouse pancreatic islets [16], the mechanism of iron efflux from the pancreas has not been investigated. Mice both lacking *Cp* and carrying a mutation in the *Heph* gene have iron loading in the pancreas [17], suggesting that CP and HEPH might both be involved in pancreatic iron efflux. In this study, we used *Heph* and *Cp* single-knockout (KO) mice and *Heph/Cp* double-knockout mice to examine the role of these MCFs in the pancreas.

## 2 Materials and methods

### 2.1 Mouse models

All animal studies were carried out in accordance with NIH guidelines. All mice used in this study were on a C57BL/6J genetic background. *Heph* KO, *Cp* KO, and wild-type (WT) mice used in this study were obtained from the laboratory of Gregory Anderson (QIMR Berghofer Medical Research Institute) as previously described [18]. *Heph* KO mice were bred to *Cp* KO mice to generate homozygous *Heph/Cp* KO mice. The mice were allowed unlimited access to a standard rodent diet containing approximately 180 mg/kg iron. All mice were bred and maintained at the Medical School of Nanjing University and the studies were approved by the Institutional Animal Care and Use Committee of Nanjing University [18]. Male mice at 6 months of age were used for all experiments.

### 2.2 Tissue collection and processing

Male mice were euthanized at six months of age. Blood was collected by cardiac puncture and the body was perfused with phosphate buffered saline (PBS) via the heart. The pancreas was quickly removed and a piece of the pancreatic tail was immediately fixed with isoamyl alcohol for later examination by transmission electron microscopy. Whole blood was centrifuged to provide plasma. Tissue samples were snap frozen in liquid nitrogen and then stored at − 80 ℃ until they were required for RNA, protein, and iron concentration analyses. In addition, four mice of each genotype were perfused via the heart, first with PBS and then with 4% paraformaldehyde. The collected tissues were fixed in 4% paraformaldehyde solution for later histological analysis.

### 2.3 Histology

Perls’ Prussian blue staining was performed as previously described [6]. TUNEL staining (Cat#11684817910, Roche) was performed following the manufacturer's protocol. For CD68 and insulin staining, paraffin-embedded pancreas sections were deparaffinized in xylenes, rehydrated in a series of ethanol rinses from 100% to 70% ethanol, then washed in distilled water. Antigen retrieval was performed at 95 °C for 30 min. Sections were allowed to cool slowly, washed in distilled water, and incubated in 3% H_2_O_2_ for 25 min. Subsequently the sections were blocked in blocking buffer containing 3% BSA and 0.1% Tween-20 in PBS (PBST), at room temperature for 30 min. Sections were stained with the primary antibody overnight at 4 °C. Then, excess antibody was removed and sections were washed 3 times with PBST for 5 min each. Sections were incubated with the secondary antibody for 50 min at room temperature. After extensive washing with PBST (three times for 5 min), the sections were washed for 5 min in PBS and incubated with diaminobenzidine substrate (Cat#K5007, Dako) to visualize the antibody. Finally, the sections were dehydrated, counterstained with hematoxylin and mounted with xylene mounting media.

For CD68 staining, a rabbit polyclonal antibody against CD68 (Cat#ab125212, Abcam; diluted 1:2000 in blocking buffer) and a horseradish peroxidase conjugated goat anti-rabbit secondary antibody (Cat#G1210-2-A, Servicebio; diluted 1:1000 in blocking buffer) were used. For insulin staining, a mouse monoclonal antibody against insulin (Cat#ab6995, Abcam; diluted 1:100 in blocking buffer) and a horseradish peroxidase conjugated goat anti-mouse secondary antibody (Cat#BA1050, Boster; diluted 1:1000 in blocking buffer) were used.

### 2.4 Measurement of tissue non-heme iron levels

The concentration of non-heme iron was measured using a microtiter plate reader as previously described [6].

### 2.5 Transmission electron microscopy

Samples of the pancreas were fixed with isoamyl alcohol and delivered to the Department of Pathology, Nanjing General Hospital for further processing. Ultrathin sections (80 nm) were cut onto copper grids, treated with the contrast agents 2% uranyl acetate and lead citrate, and examined in a JEM-1011 microscope (JEOL Ltd., Japan) with an accelerating voltage of 100 keV. Photomicrographs were analyzed with respect to the intracellular insulin granules by a histologist who was experienced in transmission electron microscopy.

### 2.6 Western blotting

Protein lysates were prepared and subjected to western blot analysis as previously described [19]. The following primary antibodies were used: anti-CP (rabbit polyclonal antibody; 1:1000; Cat#AP7340a, Abgent), anti-HEPH (rabbit polyclonal antibody raised against an N-terminus oligopeptide of HEPH; 1:1000) [20], anti-ferritin light chain (mouse monoclonal antibody; 1:1000; Cat#sc-74513, Santa Cruz Biotechnology), and anti-*β*-tubulin (mouse monoclonal antibody; 1:5000; Cat#M20005, Abmart, Shanghai, China). The levels of individual protein bands were quantified using ImageJ software (NIH) following densitometry.

### 2.7 Total RNA extraction and quantitative real-time PCR analysis

Total RNA from the tissues was isolated and reverse transcribed as previously described [18]. Real-time PCR was performed using FastStart Universal SYBR Green Master (Rox) (Cat#04913914001, Roche Applied Science) in an Applied Biosystems 7300 Real-Time PCR System machine (Life Technologies, Shanghai, China) as per the manufacturer's instructions. The levels of mRNA were normalized to that of the housekeeping gene *GAPDH*. The primer sequences used are listed in Table S1.

### 2.8 Lipase and trypsin measurements

Snap-frozen plasma was used to determine lipase and trypsin levels spectrophotometrically using a lipase assay kit (Cat#A054, Nanjing Jiancheng Bioengineering Institute, Nanjing, China) and a trypsin assay kit (Cat#A080-2, Nanjing Jiancheng Bioengineering Institute, Nanjing, China) respectively. For both analytes, data were collected using a microplate reader (Molecular Devices).

### 2.9 Analysis of oxidative stress-related markers

Lysates of snap-frozen pancreas were used to determine total superoxide dismutase (SOD) activity, total glutathione peroxidase (GPx) activity, and malonaldehyde (MDA) concentration with the following kits: Total Superoxide Dismutase Assay Kit with WST-8 (Cat#S0101; Beyotime Biotechnology); Total Glutathione Peroxidase Assay Kit (Cat#S0058; Beyotime Biotechnology); and Lipid Peroxidation MDA Assay Kit (Cat#S0131; Beyotime Biotechnology). Levels of carbonylated protein in pancreatic homogenates were measured using a Protein Carbonyl Colorimetric Assay Kit (Cat#10005020; Cayman Chemical). For all four analytes, data were collected using a microplate reader (Molecular Devices).

### 2.10 Statistical analysis

Data are shown as mean ± SEM. Statistical analysis was performed using one-way ANOVA followed by Tukey's test for multiple comparisons using GraphPad Prism 7 Software (GraphPad Software, San Diego, CA). *P* < 0.05 was considered significant.

## 3 Results

### 3.1 CP and HEPH expression in the pancreas

**Fig. 1:**
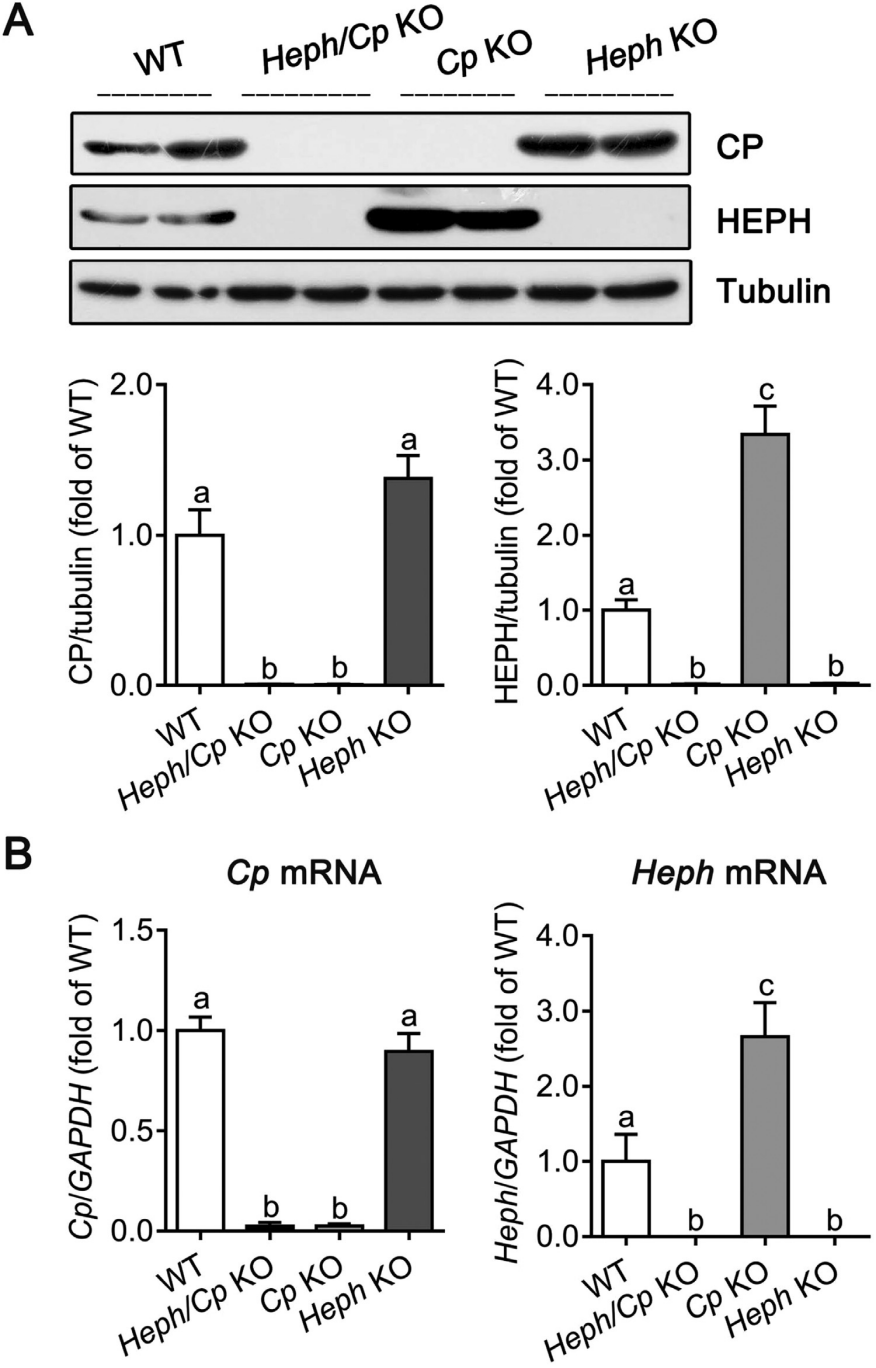
CP and HEPH protein and mRNA expression in the pancreas. (A) Expression of CP and HEPH protein in lysates from the pancreas of WT, *Heph/Cp* KO, *Cp* KO, and *Heph* KO mice (n = 4 per group). Tubulin was used as a loading control. The western blot signals were quantified, and values were normalized to tubulin expression and expressed as a proportion of the WT control. (B) Quantitative real-time PCR analysis of *Cp* and *Heph* mRNA expression in the pancreas of WT, *Heph/Cp* KO, *Cp* KO, and *Heph* KO mice (n = 6 per group). Values were normalized to *GAPDH* expression and expressed as a proportion of the WT control value. Data are shown as mean ± SEM. Bars without common letters are significantly different (p < 0.05, as determined by one way ANOVA followed by Tukey's multiple comparisons test).

We first demonstrated that both CP and HEPH proteins were abundantly expressed in the pancreas of WT mice, and that no CP or HEPH protein was detected in the corresponding gene KO mice as expected (Fig. 1A). CP protein expression was similar to the WT level in *Heph* KO mice, while the level of HEPH protein was significantly elevated (340% of WT) in *Cp* KO mice. Consistent with the protein analysis, *Cp* mRNA expression was not altered in the *Heph* KO mice compared with WT controls, whereas the *Heph* mRNA was markedly elevated (270% of WT) in *Cp* KO mice.

### 3.2 Loss of CP and HEPH induces pancreatic iron overload and oxidative damage

**Fig. 2:**
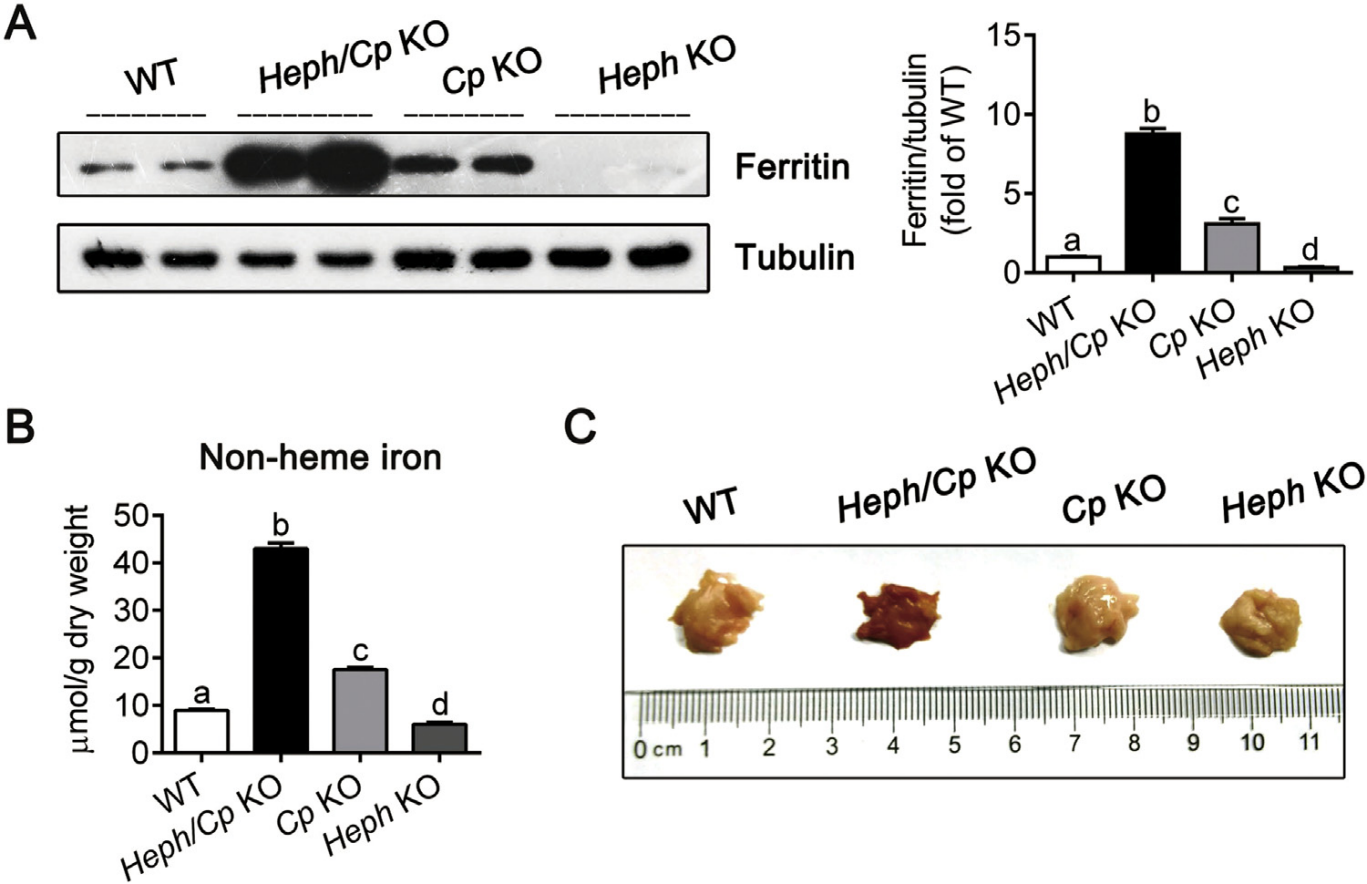
*Heph/Cp* KO mice exhibit severe iron overload in the pancreas. (A) Expression of ferritin protein in lysates from the pancreas of WT, *Heph/Cp* KO, *Cp* KO, and *Heph* KO mice (n = 4 per group). Tubulin was used as a loading control. The western blot signals were quantified, and values were normalized to tubulin expression and expressed as a proportion of the WT control value. (B) Non-heme iron concentration of the pancreas of WT, *Heph/Cp* KO, *Cp* KO, and *Heph* KO mice (n = 6 per group). (C) Macroscopic appearance of the pancreas of WT, *Heph/Cp* KO, *Cp* KO, and *Heph* KO mice. Data are shown as mean ± SEM. Bars without common letters are significantly different (p < 0.05, as determined by one way ANOVA followed by Tukey's multiple comparisons test) (For interpretation of the references to color in this figure, the reader is referred to the web version of this article.).

To evaluate the effect of CP and HEPH ablation on iron status in the pancreas, ferritin protein expression and non-heme iron were determined (Fig. 2A and B). *Heph/Cp* KO mice displayed the highest levels of ferritin and non-heme iron (900% and 500% of WT respectively), significantly higher than the other models. *Cp* KO mice also displayed higher ferritin and non-heme iron (300% and 200% of WT respectively) relative to WT control mice, but these levels were significantly lower than those of *Heph/Cp* KO mice. The lowest ferritin and non-heme iron levels were found in *Heph* KO mice (30% and 70% of WT respectively), significantly lower than those of WT mice. Moreover, similar to early studies of mice with a hepcidin-resistant *Fpn1* mutation [16], we observed a dark brown coloration in the pancreas of *Heph/Cp* KO mice, consistent with severe iron deposition (Fig. 2C).

Oxidative stress in the pancreas was assessed by measuring total SOD and total GPx activities, MDA concentration, and carbonylated protein levels (Table 1). Compared with WT mice, *Heph/Cp* KO and *Cp* KO mice displayed significantly lower SOD activities (35% and 70% of WT respectively), but no significant difference was noted in *Heph* KO mice. GPx activity was also significantly decreased in *Heph/Cp* KO mice (30% of WT), but not in *Cp* KO or *Heph* KO mice. The MDA concentration and protein carbonyls were significantly increased in *Heph/Cp* KO mice (500% and 140% of WT respectively), but no alterations were observed in the single KO mouse strains. Together, these results are consistent with severe iron deposition in the pancreas of *Heph/Cp* KO mice leading to significant oxidative damage.

**Table 1:** Total SOD and total GPx activities, MDA concentrations, and protein carbonyls in the pancreas of WT, *Heph/Cp* KO, *Cp* KO, and *Heph* KO mice at 6 months of age*.

Oxidative stress markers	WT	*Heph/Cp* KO	*Cp* KO	*Heph* KO
Total SOD activity (U/mg protein)	23 ± 1.3^a^	8 ± 1.0^b^	16 ± 1.3^c^	22 ± 1.3^a^
Total GPx activity (U/mg protein)	6.9 ± 0.6^a^	2.0 ± 0.2^b^	6.7 ± 0.5^a^	7.1 ± 0.7^a^
MDA concentration (nmol/mg protein)	0.5 ± 0.1^a^	2.6 ± 0.3^b^	0.7 ± 0.1^a^	0.5 ± 0.1^a^
Protein carbonyls (nmol/mg protein)	1.2 ± 0.1^a^	1.7 ± 0.1^b^	1.3 ± 0.1^a^	1.2 ± 0.1^a^

Results without common letters are significantly different, p < 0.05. SOD, superoxide dismutase; GPx, glutathione peroxidase; MDA, malondialdehyde; WT, wild-type; *Heph/Cp* KO, hephaestin and ceruloplasmin double knockout; *Cp* KO, ceruloplasmin knockout; *Heph/Cp* KO, hephaestin knockout.

* Values represent the mean ± SEM, n = 6.

### 3.3 Pancreatic expression of mRNAs encoding iron-related proteins and proinflammatory cytokines

**Fig. 3:**
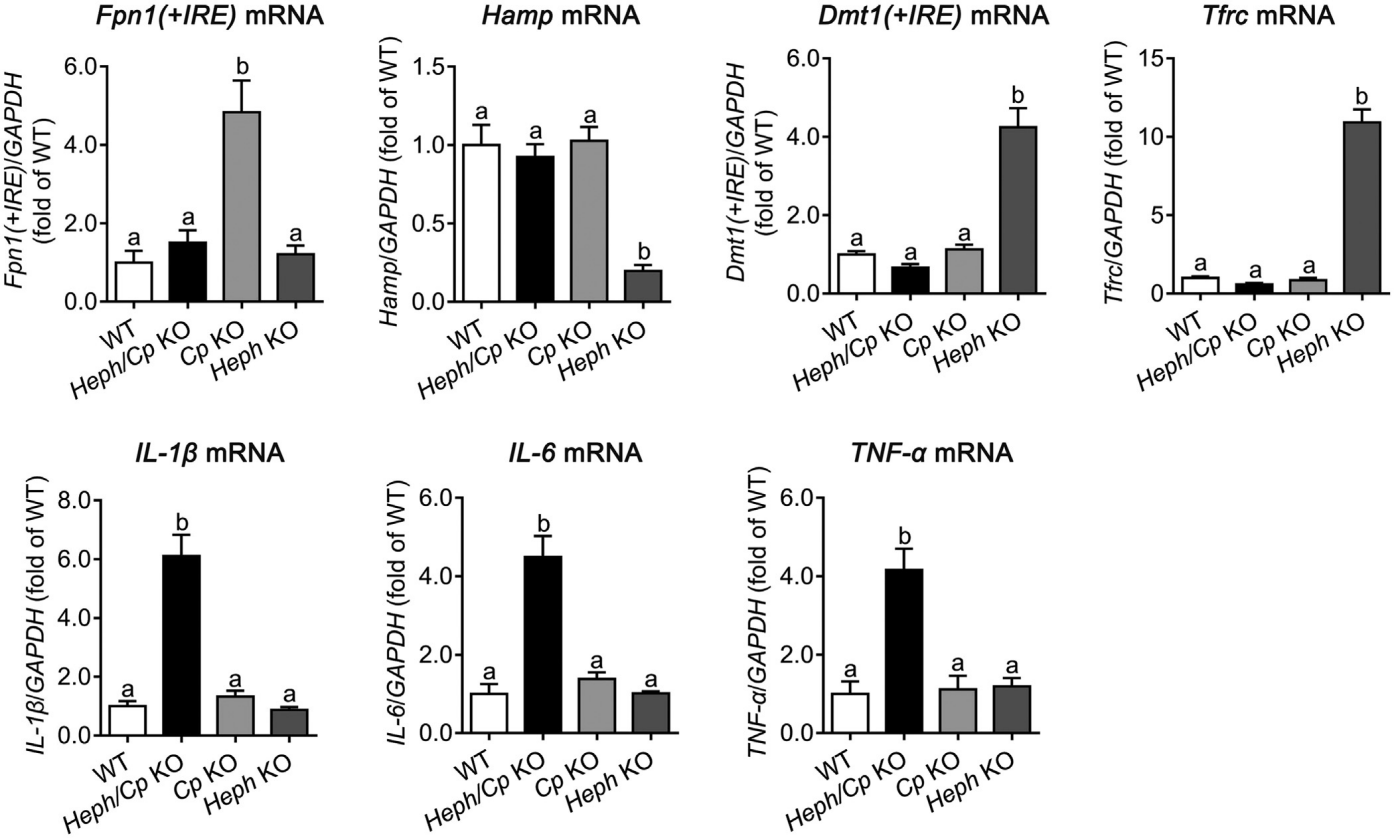
Expression of the mRNAs encoding iron-related proteins and proinflammatory cytokines in the pancreas. *Fpn1(+IRE)*, *Hamp*, *Dmt1(+IRE)*, *Tfrc*, *IL-1β*, *IL-6*, and *TNF-α* mRNA expression in the pancreas of WT, *Heph/Cp* KO, *Cp* KO, and *Heph* KO mice is shown (n = 6 per group). Values were normalized to *GAPDH* expression and expressed as a proportion of the WT control value. Data are shown as mean ± SEM. Bars without common letters are significantly different (p < 0.05, as determined by one way ANOVA followed by Tukey's multiple comparisons test).

*Fpn1(+iron responsive element (IRE))*, *Hamp* (combined *Hamp1* and *Hamp2*), *Dmt1(+IRE)*, *Tfrc*, *IL-1β*, *IL-6*, and *TNF-α* mRNA levels in the pancreas were examined by quantitative real-time PCR (Fig. 3). A significantly increased level of *Fpn1(+IRE)* mRNA was noted in *Cp* KO mice (500% of WT), but no significant differences were observed in the other genotypes. *Hamp*, *Dmt1(+IRE)*, or *Tfrc* mRNA did not significantly change in *Cp* KO or *Heph/Cp* KO mice, but in *Heph* KO mice, *Hamp* expression was significantly decreased (20% of WT), and *Dmt1(+IRE)* and *Tfrc* mRNAs were significantly increased (400% and 1100% of WT respectively) relative to WT control mice. The mRNAs encoding the proinflammatory cytokines *IL-1β*, *IL-6*, and *TNF-α* were significantly increased (600%, 450% and 400% of WT respectively) in *Heph/Cp* KO mice, but no significant differences were observed in *Cp* KO or *Heph* KO mice.

### 3.4 Iron deposition predominated in the exocrine pancreas of Heph/Cp KO mice

**Fig. 4:**
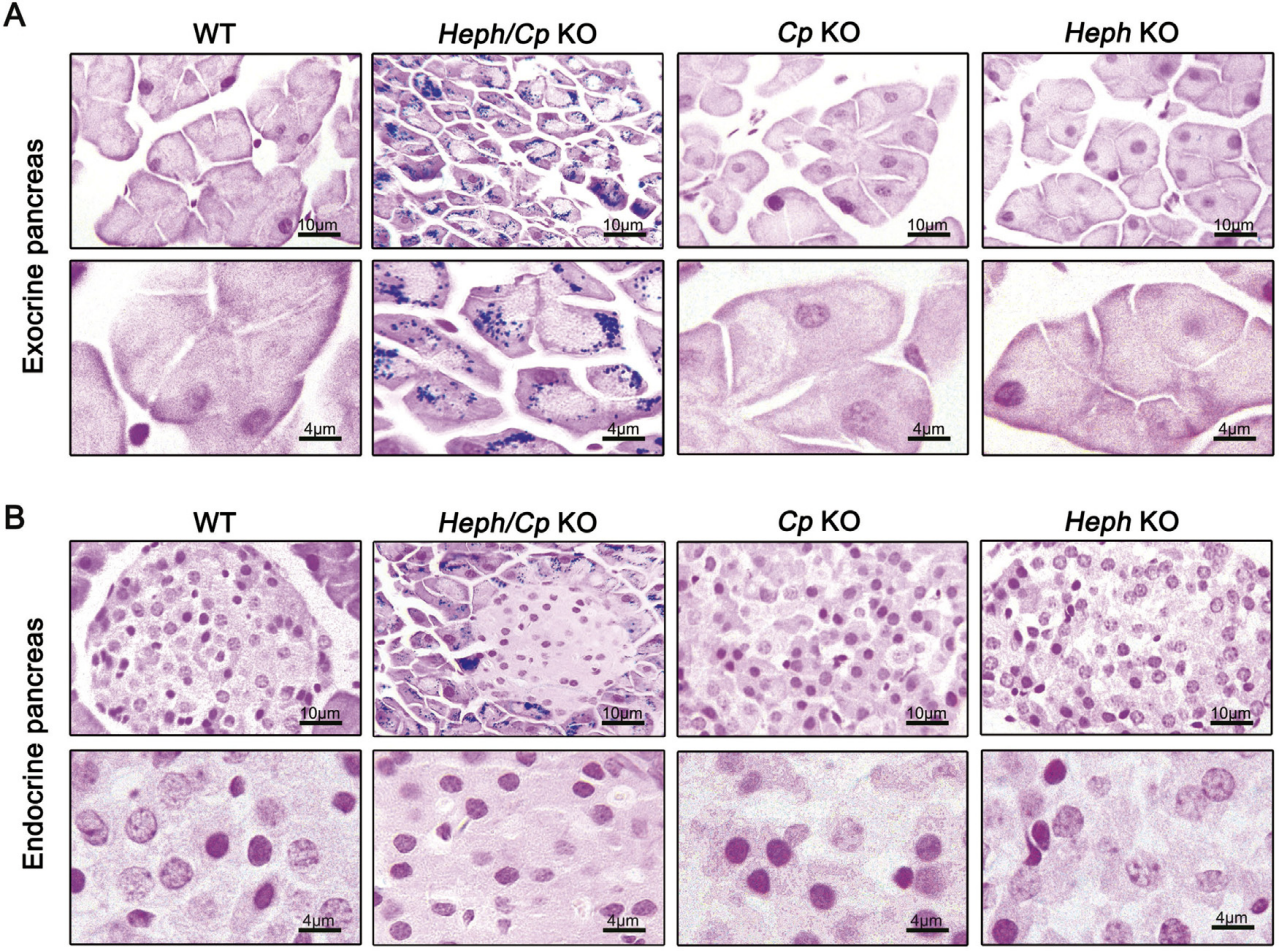
Iron deposition predominates in the exocrine pancreas of *Heph/Cp* KO mice. (A and B) Representative images of Perls’ Prussian blue staining for iron in the exocrine (A) and endocrine (B) pancreas of WT, *Heph/Cp* KO, *Cp* KO, and *Heph* KO mice (n = 4 per group). In each part of the figure, the upper row is of lower magnification than the lower row.

Iron distribution in the pancreas was assessed histologically using Perls’ Prussian blue staining. At a lower magnification (400×), severe iron deposition was apparent in the exocrine pancreas of *Heph/Cp* KO mice, but no iron was observed in the other models (Fig. 4A, top). At higher magnification (1000 ×), iron deposition was apparent in the acinar cells in *Heph/Cp* KO mice, suggesting disrupted iron efflux from these cells (Fig. 4A, bottom). In addition, similar to hepcidin KO mice and hypotransferrinaemic mice [15], [21], acinar atrophy was observed in the *Heph/Cp* KO mice, as the size of the acinar cells was clearly smaller than in the other models. Interestingly, in contrast to the exocrine pancreas, the endocrine pancreas of *Heph/Cp* KO mice was iron spared. No iron was observed in the endocrine pancreas in any of the other mouse models (Fig. 4B).

### 3.5 Heph/Cp KO mice show exocrine pancreatic injury and inflammatory infiltration

**Fig. 5:**
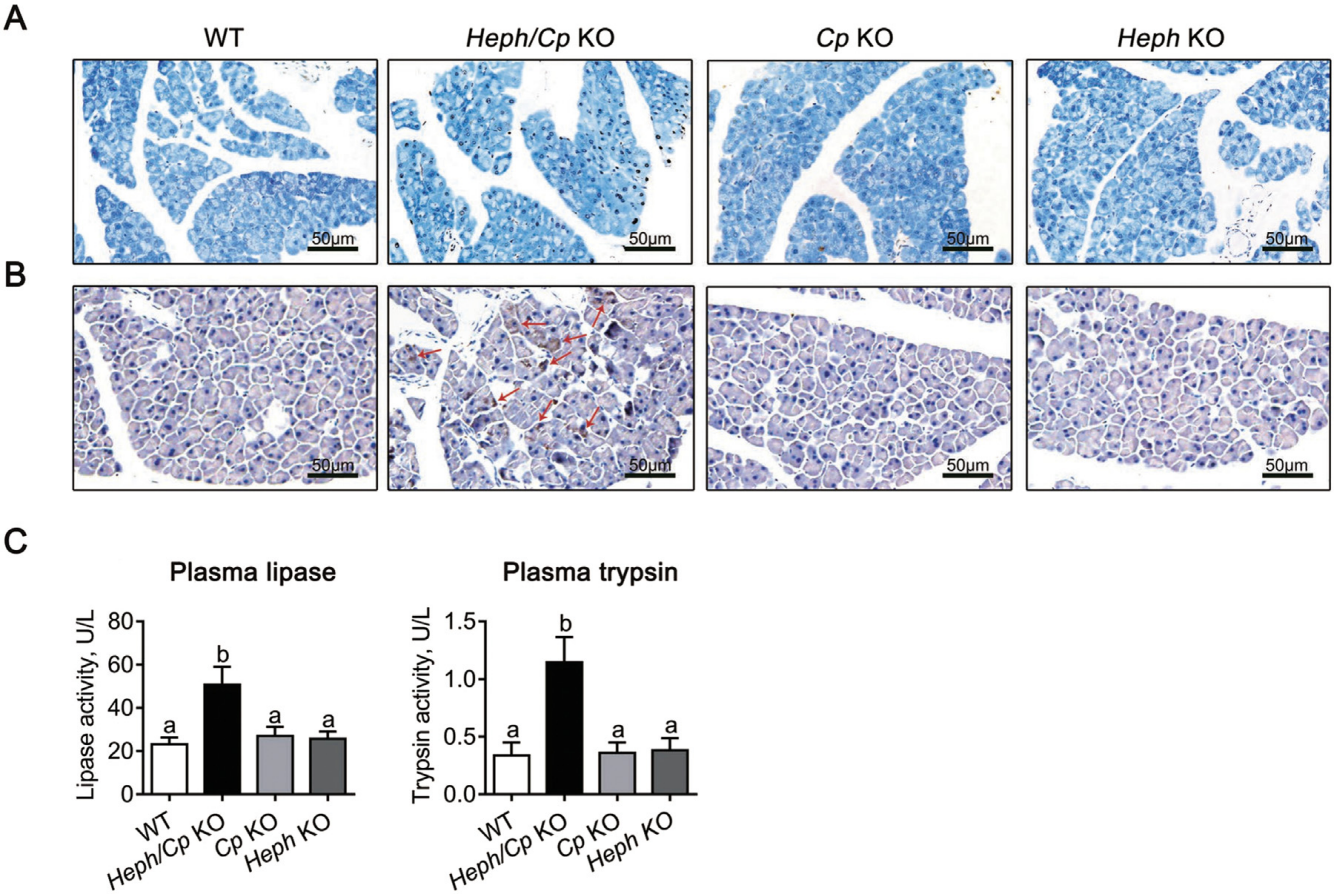
*Heph/Cp* KO mice exhibit manifestations of exocrine pancreatic injury and inflammatory infiltration. (A and B) Representative images of TUNEL staining for apoptotic cells (A) and CD68 staining for macrophages (B) in the exocrine pancreas of WT, *Heph/Cp* KO, *Cp* KO, and *Heph* KO mice (n = 4 per group). (C) Plasma lipase and trypsin activities of WT, *Heph/Cp* KO, *Cp* KO, and *Heph* KO mice (n = 6 per group). Data are shown as mean ± SEM. Bars without common letters are significantly different (p < 0.05, as determined by one way ANOVA followed by Tukey's multiple comparisons test).

Apoptosis is an important marker of pancreatic injury [15] and was assessed by TUNEL staining. Similar to early studies of hepcidin KO mice [15], significant increase in the number of apoptotic cells was observed in the exocrine pancreas of *Heph/Cp* KO mice (Fig. 5A). Inflammatory infiltration was assessed by immunostaining for the macrophage marker CD68, and the number of CD68-positive cells in the exocrine pancreas of *Heph/Cp* KO mice was much more than that in the other groups (Fig. 5B). Plasma lipase and trypsin are typically elevated when the exocrine pancreas is dysfunctional [22], and we found them to be significantly increased (220% and 340% of WT respectively) in *Heph/Cp* KO mice. No alterations were observed in *Heph* KO or *Cp* KO mice (Fig. 5C). Together, these results suggest that the exocrine pancreas of *Heph/Cp* KO mice is significantly damaged by the accumulated iron.

**Fig. 6:**
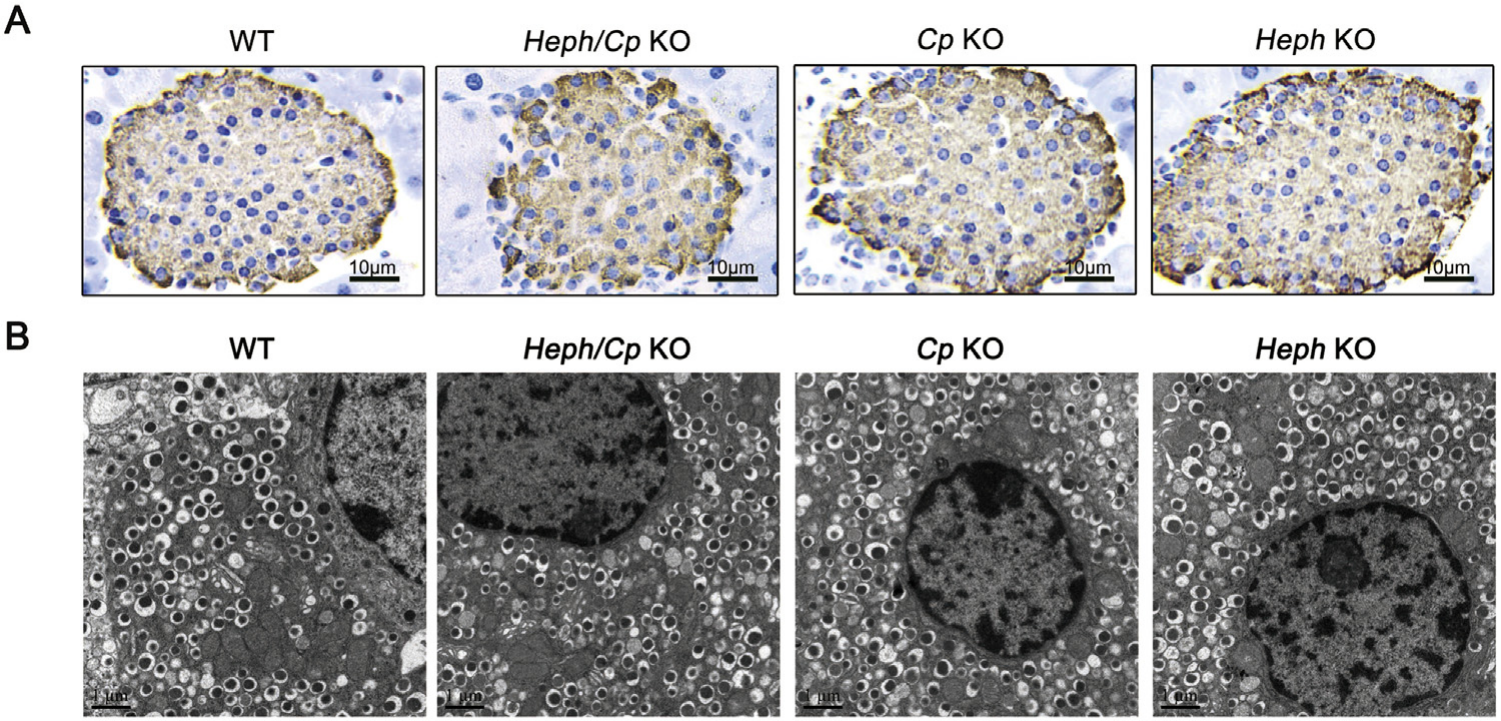
Insulin levels are not altered in *Heph/Cp* KO mice. (A) Representative images of insulin staining in islets of WT, *Heph/Cp* KO, *Cp* KO, and *Heph* KO mice (n = 4 per group). (B) Transmission electron micrographs of insulin granules in *β-*cells of WT, *Heph/Cp* KO, *Cp* KO, and *Heph* KO mice (n = 4 per group).

Insulin secretion is a basic function of the endocrine pancreas; thus we performed insulin immunostaining and transmission electron microscopy to determine whether the endocrine pancreas was affected by MCF deletion. There were no significant differences in insulin staining among the four models studied (Fig. 6A). Consistent with this, transmission electron microscopy also revealed no decline in the number of insulin granules in the *β*-cells of *MCF* KO mice (Fig. 6B).

## 4 Discussion

HEPH is most strongly expressed in the intestine and most CP is secreted by the liver. However, recent studies have shown that HEPH and CP are co-expressed in many tissues (e.g. kidney, adipose tissue, brain) [19], [23], [24], [25]. Ablation of HEPH and CP globally induces significant iron accumulation in these tissues, and leads to dramatically lower plasma iron and anemia [19], [23], [24], [25]. Our recent work has demonstrated the mutually compensatory roles of HEPH and CP in adipose tissue, and how loss of both MCFs results in adipocyte iron deposition, insulin resistance and type 2 diabetes [24]. Notably, *Heph/Cp* KO mice display a significantly increased plasma insulin level, indicating that the insulin secretion was not impaired [24]. In this study, we focus on the pancreas and investigate the roles of HEPH and CP in pancreatic iron metabolism.

Previous studies have reported that patients with hereditary ceruloplasmin deficiency display iron deposition in the exocrine pancreas [10], [26], [27], and one study identified the expression of GPI-anchored *Cp* mRNA in the pancreas [28]. HEPH, however, has only been reported in one study to be expressed in human pancreatic islets [7]. Here, we first used western blotting to show that both CP and HEPH proteins were expressed in the pancreas (Fig. 1A). Consistent with a previous report in humans lacking CP [10], our *Cp* KO mice showed iron loading in the pancreas, while *Heph* KO mice were iron deficient. These findings are not surprising considering what we know about these proteins. CP, for example, is involved in iron efflux from multiple internal organs, so when it is missing, iron which enters these tissues is not recycled to the plasma efficiently and is retained within the cells. This effectively leads to a redistribution of iron from the plasma to the tissues [1]. In contrast, ablation of HEPH directly decreases iron absorption from the intestine and results in systemic iron deficiency [6]. In *Heph/Cp* KO mice, although intestinal iron absorption was also impaired (data not shown), the pancreas was still iron overloaded (much more severe than *Cp* KO mice), suggesting that both HEPH and CP are involved in pancreatic iron efflux and that they have mutually compensatory roles in this process.

CP and HEPH likely work in concert with FPN1 to facilitate efficient cellular iron efflux [1]. In the pancreas of *Cp* KO mice, HEPH protein, *Heph* mRNA, and *Fpn1(+IRE)* mRNA were significantly increased, suggesting a compensatory increase in the absence of CP to help reduce the tissue iron load. However, in the pancreas of *Heph* KO mice, no alterations were observed in CP protein, *Cp* mRNA, or *Fpn1(+IRE)* mRNA, although in this situation there would be no particular drive to increase cellular iron efflux from this organ, as the pancreas was iron-deficient. Interestingly, *Fpn1(+IRE)* mRNA levels were not increased in *Heph/Cp* KO mice, despite the severe pancreatic iron deposition. Perhaps this reflects some undiscovered feedback mechanism, where lack of MCFs leads to FPN1 transcript downregulation. These data also suggest that iron status is not the only factor influencing *Fpn1(+IRE)* mRNA expression in this tissue. *Hamp*, *Dmt1(+IRE)*, and *Tfrc* mRNA expression, curiously, were only changed in *Heph* KO mice. In broad terms, these changes are consistent with the pancreas protecting itself against iron deficiency, but why these genes were not altered when the pancreas was iron loaded is unclear.

Iron overload in the exocrine pancreas might be expected to be associated with the generation of ROS and the development of pancreatitis [15], [16]. Iron-loaded acinar cells are a potent driving force for oxidative stress as they release H_2_O_2_, which may undergo the Fenton reaction and cause the generation of the highly toxic hydroxyl radical [29]. Increased MDA and protein carbonyls are markers of lipid peroxidation and protein oxidation respectively, and SOD and GPx are endogenous scavengers that provide defense against ROS [30], [31]. The dramatic elevation of MDA and protein carbonyl levels, accompanied by significant decline of total SOD and total GPx activities (Table 1), indicates severe oxidative damage in the pancreas of *Heph/Cp* KO mice. This could partly (if not entirely) explain the exocrine pancreatic injury in the double KO mice. A previous study has showed that hepcidin KO mice developed pancreatitis at 6 months of age due to cytoplasmic iron overload in acinar cells [15]. Likewise, the simultaneous deficiency in ceruloplasmin and hephaestin leads to inflammatory infiltration and up-regulation of pro-inflammatory cytokines in exocrine pancreas, and increased activities of lipase and trypsin in plasma. These data suggest that iron accumulation in acinar cells elicits pancreatitis in *Heph/Cp* KO mice.

In contrast to the exocrine pancreas, ablation of both MCFs had little effect on the endocrine pancreas, and these results are also in accordance with our previous observation that plasma insulin was not decreased in *Heph/Cp* KO mice [24]. In the pancreas of hemochromatosis patients, large amounts of iron are found in the exocrine pancreas, but occasionally there is some iron in the islets [32]. Likewise, patients with hereditary ceruloplasmin deficiency display much milder iron accumulation in the islets compared with the exocrine pancreas [10], [27]. In mouse models of hemochromatosis, the endocrine pancreas is generally unaffected [15], [16], [33]. Although the reasons for these patterns of iron deposition require further investigation, this work suggests that there might be other iron efflux pathways independent of HEPH and CP in the endocrine pancreas, protecting it against iron accumulation and oxidative damage.

In conclusion, our results suggested that CP and HEPH play important and mutually compensatory roles in facilitating iron efflux from the exocrine pancreas and preventing oxidative damage. They also suggested that HEPH and CP are not essential for iron homeostasis in the endocrine pancreas. To better understand the mechanism of pancreatic iron metabolism, additional research is needed.
