# Supplementary material for: Ceruloplasmin and hephaestin jointly protect the exocrine pancreas against oxidative damage by facilitating iron efflux
Source: Redox Biol. 2018 May 31;17:432–9. doi: 10.1016/j.redox.2018.05.013 (PMC6007082; doi:10.1016/j.redox.2018.05.013)
Supplement: Supplementary file 1 — Supplementary material [file mmc1.docx]

**Supplementary material**

**Table S1**

Primers for quantitative real-time PCR.

|  | Primer forward | Primer reverse |
| --- | --- | --- |
| *Cp* | TCTACCAAGGAGTAGCCAGGA | ATCTTCCCTCTCATCCGTGC |
| *Heph* | GAATTTTGCGAGCCGACCTT | TCATCCGCTTTCAGATACCC |
| *Fpn1(+IRE)* | TCGGTTCCTCTCACTCCTGT | GTGGAGAGAGAGTGGCCAAG |
| *Hamp* | AGAGCTGCAGCCTTTGCAC | ACACTGGGAATTGTTACAGCATTTA |
| *Dmt1(+IRE)* | TAGGCTGTGCTCAAACCTACAGCA | TACATGAGAGCCAGGCATGGTAGA |
| *Tfrc* | GGTGTTGCGGCGAAGTCCAGT | ACTCAGTGGCACCAACAGCTCC |
| *IL-1β* | ACGGACCCCAAAAGATGAAG | TTCTCCACAGCCACAATGAG |
| *IL-6* | AGGAGACTTCACAGAGGATACC | GAATTGCCATTGCACAACTCTT |
| *TNF-α* | TCCCAGGTTCTCTTCAAGGGA | GGTGAGGAGCACGTAGTCGG |
| *GAPDH* | AACTTTGGCATTGTGGAAGG | GGATGCAGGGATGATGTTCT |

Cp, ceruloplasmin; Heph, hephaestin; Fpn1(+IRE), ferroportin1 (with iron responsive element); Hamp, hepcidin antimicrobial peptide; Dmt1(+IRE), divalent metal transporter 1 (with iron responsive element); Tfrc, transferrin receptor 1; IL-1β, interleukin-1 beta; IL-6, interleukin-6; TNF-α, tumor necrosis factor-alpha; GAPDH, glyceraldehyde-3-phosphate dehydrogenase.
